# Three dimensional first-pass myocardial perfusion imaging at 3T: feasibility study

**DOI:** 10.1186/1532-429X-10-57

**Published:** 2008-12-11

**Authors:** Taehoon Shin, Houchun H Hu, Gerald M Pohost, Krishna S Nayak

**Affiliations:** 1 Ming Hsieh Department of Electrical Engineering, University of Southern California, Los Angeles, California, USA; 2 Keck School of Medicine, University of Southern California, Los Angeles, California, USA

## Abstract

**Background:**

In patients with ischemic heart disease, accurate assessment of the extent of myocardial perfusion deficit may be important in predicting prognosis of clinical cardiac outcomes. The aim of this study was to compare the ability of three dimensional (3D) and of two dimensional (2D) multi-slice myocardial perfusion imaging (MPI) using cardiovascular magnetic resonance (CMR) in determining the size of defects, and to demonstrate the feasibility of 3D MPI in healthy volunteers at 3 Tesla.

**Methods:**

A heart phantom was used to compare the accuracy of 3D and 2D multi-slice MPI in estimating the volume fraction of seven rubber insets which simulated transmural myocardial perfusion defects. Three sets of cross-sectional planes were acquired for 2D multi-slice imaging, where each set was shifted along the partition encoding direction by ± 10 mm. 3D first-pass contrast-enhanced (0.1 mmol/kg Gd-DTPA) MPI was performed in three volunteers with sensitivity encoding for six-fold acceleration. The upslope of the myocardial time-intensity-curve and peak SNR/CNR values were calculated.

**Results:**

Mean/standard deviation of errors in estimating the volume fraction across the seven defects were -0.44/1.49%, 2.23/2.97%, and 2.59/3.18% in 3D, 2D 4-slice, and 2D 3-slice imaging, respectively. 3D MPI performed in healthy volunteers produced excellent quality images with whole left ventricular (LV) coverage. Peak SNR/CNR was 57.6 ± 22.0/37.5 ± 19.7 over all segments in the first eight slices.

**Conclusion:**

3D performed better than 2D multi-slice MPI in estimating the size of perfusion defects in phantoms. Highly accelerated 3D MPI at 3T was feasible in volunteers, allowing whole LV coverage with excellent image quality and high SNR/CNR.

## Background

Ischemic heart disease (IHD) is a leading cause of mortality in the world, accounting for over 7 million deaths in 2002 and over 9 million expected deaths by the year 2030 [1]. Coronary catheter-based X-ray angiography (coronary angiography) is currently the "gold standard" modality for assessing disease of the coronary arteries that is the basis for IHD. However, coronary angiography is an invasive approach requiring insertion of a catheter into the coronary artery and administration of contrast agent to demonstrate the coronary artery disease. Considerable attention has been paid to non-invasive myocardial perfusion imaging methods that provide a means to assess the distribution of blood flow within the myocardium. Presently, single photon emission computed tomography (SPECT) is routinely used in clinical myocardial perfusion imaging (MPI) studies, but suffers from attenuation artifacts, low spatial resolution and exposure to ionizing radiation [2].

First-pass cardiovascular magnetic resonance (CMR) MPI is an ideal tool for the evaluation of myocardial perfusion, due to its high spatial resolution, lack of ionizing radiation, and relatively short scan time. First-pass CMR MPI tracks the passage of a gadolinium-based T1 contrast agent by time resolved T1 weighted imaging [3]. Two dimensional (2D) multi-slice imaging is typically used as a means for data acquisition in CMR MPI, and has demonstrated high sensitivity (>85%) and specificity (>75%) [4-7]. Besides its diagnostic utility, MPI is also an important approach for assessment of prognosis in symptomatic patients. Patient follow-up studies have shown that the extent of abnormal myocardial perfusion is useful for the prediction of cardiac outcomes [8-10]. However, its accurate estimation is restricted in conventional 2D multi-slice MPI, due to the limited tradeoff between spatial coverage and inter-slice spacing.

Three dimensional (3D) CMR MPI is an advantageous alternative to 2D multi-slice CMR MPI since it supports contiguous spatial coverage and therefore has the potential to more accurately estimate the extent (volume) of abnormal perfusion. Moreover, 3D encoding inherently retains high signal-to-noise ratio (SNR) efficiency and high capacity for parallel imaging acceleration [11], and has been demonstrated at 1.5 T [12]. The purpose of the present study was (i) to evaluate the performance of 3D MPI and 2D multi-slice MPI for estimating defect size in a phantom study, and (ii) to demonstrate the feasibility of in-vivo 3D MPI accelerated by sensitivity encoding (SENSE) encompassing the entire left ventricle (LV) at 3 Tesla.

## Methods

All experiments were performed on a General Electric 3 Tesla scanner with peak gradient amplitude of 40 mT/m and peak slew rate of 150 T/m/s, using a body-coil for RF transmission and an eight-channel cardiac coil array for signal reception.

### Pulse sequence

**Figure 1:**
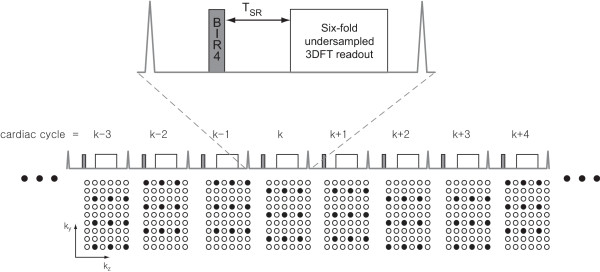
**Pulse sequence diagram and sampling pattern for 3D myocardial perfusion imaging**. The pulse sequence consists of a 90° BIR-4 adiabatic RF pulse for myocardial saturation, followed by a saturation recovery time and undersampled 3DFT GRE readout. 3D *k*-space was undersampled by factor of three and two in *ky *and in *kz*, respectively, resulting in a net undersampling factor of six. The *k*-space sampling locations were shifted in a cyclic pattern such that coil sensitivity map could be obtained from the data in six neighboring R-R intervals. SENSE reconstruction of undersampled data from the k^*th *^cardiac cycle utilized coil sensitivity maps derived from the (k-2)^*th *^to (k+3)^*th *^cardiac cycles.

The pulse sequence used in this study is a 3DFT gradient echo (GRE) acquisition that is preceded by a 90° global saturation pulse and a saturation delay time T_*SR *_(Fig. 1). We specifically adopted an adiabatic saturation pulse (BIR-4) due to its insensitivity to transmit B1 non-uniformity [13,14]. For slab selective excitation, a sinc pulse with a time-bandwidth-product (TBW) of four was used. ECG gating was used to synchronize data acquisition at diastole to minimize the effects of cardiac motion.

3D *k*-space for a given spatial resolution and FOV was undersampled at a net acceleration factor of six. Since the readout encoding line (*k*_*x*_) is always fully sampled, undersampling in the other two encoding lines (*k*_*y*_, *k*_*z*_) can be viewed in the 2D *k*_y_-*k*_z _plane (Fig. 1). Six-fold undersamped data were obtained by three-fold undersampling in *k*_y _and two-fold undersampling in *k*_*z*_. Aliasing-free 3D images were reconstructed by using SENSE, and the resulting temporal resolution was one heart beat. The position of sampled *k*-space encoding lines was shifted in a cyclic pattern in the *k*_y_-*k*_z _plane such that acquired data from six neighboring cardiac cycles could be combined to form a coil sensitivity map for SENSE reconstruction (Fig. 1).

### Phantom experiment

#### Phantom description

**Figure 2:**
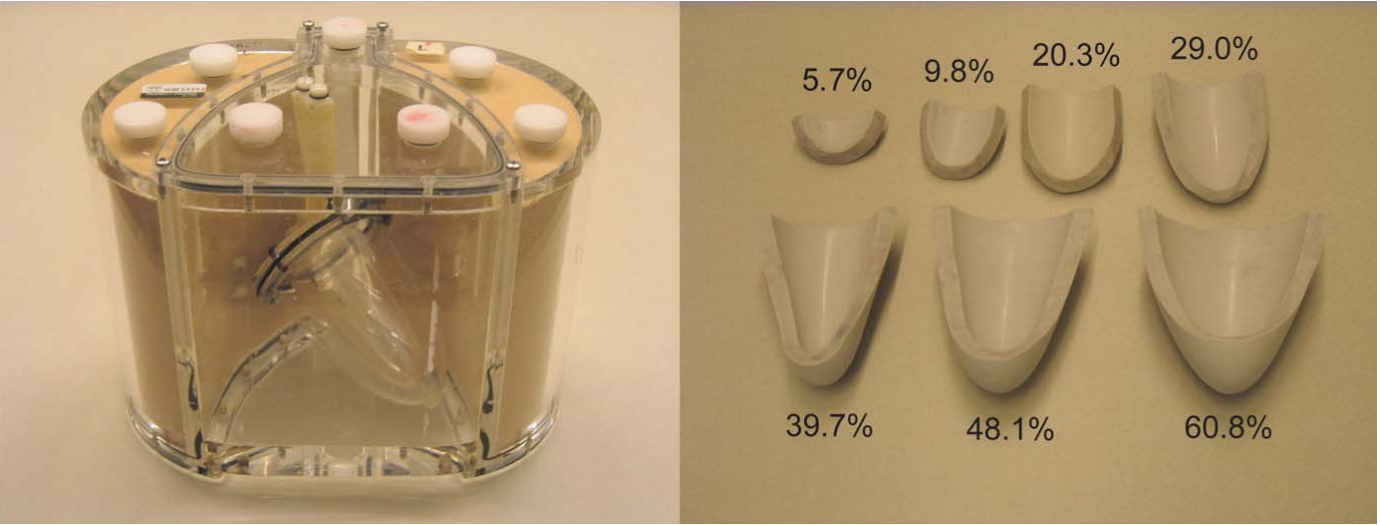
**Cardiac phantom and rubber insets**. Left ventricular and myocardial cavities were filled with Gadolinium-doped saline of 2 mmol/L and 0.6 mmol/L. The rubber insets ranging in size from 5~70% of the myocardial volume, mimic transmural perfusion defects.

A heart phantom (Model RH-2, Capintec Inc.) was scanned to compare the accuracy of 3D and 2D multi-slice MPI in estimating defect size (Fig. 2) [15]. The phantom contained left ventricular cavity of 132 mL volume and myocardial cavity of 193 mL volume, 10.5 mm thickness and 11 cm long-axis length. The two cavities were separated by a ~2 mm thick wall, and were filled with Gadolinium-doped saline of 2 mmol/L and 0.6 mmol/L, to mimic peak enhancement in LV and myocardium, respectively [16]. Seven rubber insets ranging in size from approximately 5–60% of the myocardial volume were used to mimic transmural perfusion defects.

#### Scan protocol

3D scanning was performed using the pulse sequence illustrated in Fig. 1. Full k-space data was acquired in six segments with NEX = 10, requiring 6 × 10 = 60 data acquisitions. 2D multi-slice scanning was performed using a BIR-4 saturation pulse followed by 2DFT GRE acquisition. Full k-space data was acquired in two segments per slice with NEX = 10, requiring a total of 2 × 10 = 20 data acquisitions per slice. Specific imaging parameters used for 3D and 2D 4-slice/3-slice scans are represented in Table 1. The long-axis spatial coverage was 10.0 cm, 8.2 cm and 7.0 cm in 3D, 2D 4-slice, and 2D 3-slice scans, respectively. The gap between neighboring slices was 14 mm and 20 mm in 2D 4-slice and 2D 3-slice scans, respectively. Three sets of cross-sectional scan planes each of which was shifted by 10 mm along the partition encoding direction, were used for 2D multi-slice imaging to examine the sensitivity of scan plane locations on the estimation of defect size.

**Table 1:** Imaging parameters of 3D and 2D multi-slice scans

	3D	2D (4-slice/3-slice)
T_*SR*_	100 ms	100 ms
TR	2.3 ms	2.9 ms
TE	0.9 ms	1.3 ms
flip angle	12°	12°
matrix size	100 × 66 × 10	120 × 80
FOV	30 × 30 × 10 cm^3^	30 × 30 cm^2^
slice thickness	10 mm	10 mm
slice gap	0	14/20 mm
long-axis coverage	100 mm	82/70 mm
NEX	10	10

#### Image analysis

The borders of defects in reconstructed images were manually outlined, and the volume of a defect was expressed as the percent of the whole myocardium. To accurately estimate partial inclusion of the insets in a voxel, the phantom image with a defect was normalized by the image without a defect, and the fraction of defect was linearly interpolated in each normalized voxel.

### *In-vivo *experiments

#### Scan protocol

In-vivo experiments using 3D MPI were performed in three volunteers. Written informed consent was obtained from all participants. Imaging parameters were the same as those in Table 1 except T_*SR *_= 130 ms and matrix size = 100 × 66 ~69 × 10. Data acquisition was located at the center of diastole, and the acquisition time was 304 ms. A proton density weighted data set was obtained using 4° flip angle with the saturation pulse turned off during the first six cardiac cycles. Contrast media (0.1 mmol/kg Gd-DTPA, Magnevist) was injected at a rate of 5 ml/s followed by 20 ml saline flush at the same rate. Subjects were instructed to hold their breath as long as possible.

#### Reconstruction

All image reconstruction was performed off-line using MATLAB (Mathworks, Natick, MA). Proton density weighted images were obtained by simply combining the *k*-space data from the first six cardiac cycles. SENSE was used for the image reconstruction from six-fold undersampled 3DFT perfusion data [11,17]. SENSE reconstruction of undersampled data from the k^*th*^cardiac cycle utilized coil sensitivity maps derived from the (k-2)^*th *^to (k+3)^*th *^cardiac cycles (see Fig. 1).

#### Image analysis

Raw perfusion images were normalized by proton density images to remove variations in the receiver coil sensitivities. Corrected images were then segmented into six (basal and mid-short axis levels) or four (apical level) myocardial sectors, according to the American Heart Association 17-segment model [18]. Within each myocardial sector, a time-intensity-curve (TIC) was generated and the corresponding upslope value was computed by linear fitting of the data during signal enhancement.

Noise standard deviation is typically calculated from the background region-of-interest (ROI) for the computation of SNR, assuming spatially uniform noise distribution [19,20], but this assumption is no longer valid in SENSE reconstruction due to spatially varying reconstruction noise. Hence in this work, SNR was calculated by the "difference method" [21], where noise standard deviation was estimated from the difference in two consecutive images.

## Results

### Phantom experiments

**Figure 3:**
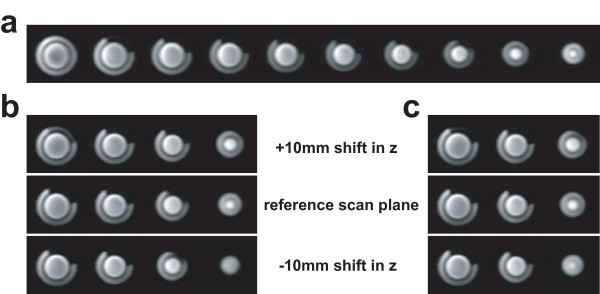
**Representative heart phantom images with a 20.3% rubber inset**. (a): images from 3D scan, (b): images from 2D 4-slice scan, and (c): images from 2D 3-slice scan. In 2D multi-slice case, images from the three sets of cross-sectional scan planes were shown. In all phantom images, the dark rim around the LV was created by the ~2 mm thick separation wall between the LV and the myocardial cavities.

**Figure 4:**
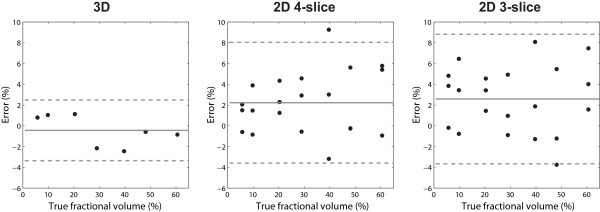
**Error plotted for increasing defect size expressed as a percent of total volume**. The horizontal axis and vertical axis represent the true volume fraction of the defect, and the estimation errors, respectively. In each plot, the solid and dashed lines represent the mean and ± 1.96 × standard deviation, respectively. 3D imaging resulted in a significantly lower bias/standard deviation (-0.44/1.49%) compared to 2D 4-slice (2.23/2.97%) and 2D 3-slice (2.59/3.18%).

Figure 3 shows representative images of the phantom with a 20.3% rubber inset obtained from 3D, 2D 4-slice and 2D 3-slice scans. Figure 4 contains scatter plots of errors in the estimation of defect size using the three methods. The horizontal axis and vertical axis represent the true volume fraction of the defect, and estimation errors, respectively. The mean and ± 1.96 × standard deviation are denoted by solid and dotted lines, respectively. In 2D cases, three measurements were made for each phantom defect from three shifted scan orientations. 3D imaging resulted in a significantly smaller bias/standard deviation (-0.44/1.49%) compared to 2D 4-slice imaging (2.23/2.97%) and 2D 3-slice imaging (2.59/3.18%). 2D multi-slice methods show a slight bias towards overestimation, presumably due to the fact that the majority of defects in this study were positioned at mid-short axis level and were partially visible in all slices. 2D multi-slice methods also show a larger measurement standard deviation due to incomplete spatial coverage.

### *In-vivo *experiments

**Figure 5:**
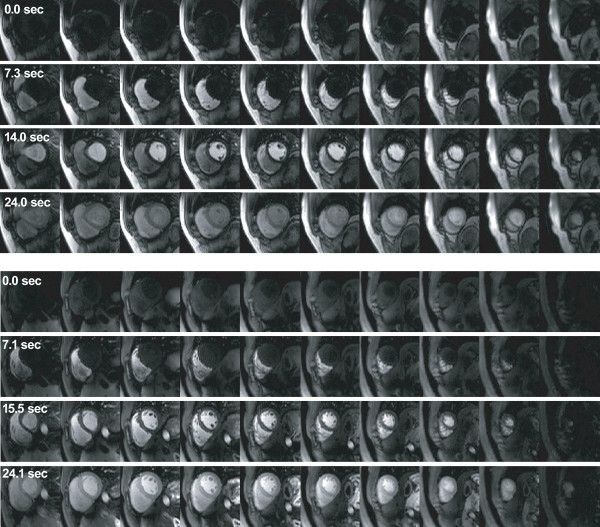
**Representative 3D perfusion images from two healthy volunteers**. The four rows in each subject show perfusion images at pre-contrast, right ventricular (RV) enhancement, left ventricular (LV) enhancement, and myocardial enhancement. Whole LV can be covered by ten partition slices with excellent image quality.

Figure 5 shows representative 3D perfusion images from one subject, at pre-contrast, RV enhancement, LV enhancement, and myocardial enhancement. Overall image quality is excellent due to high SNR and effective SENSE reconstruction, clearly showing the arrival and the passage of contrast agent. A small amount of signal fluctuation is seen during the course of RV and LV enhancement. This flickering artifact is due to errors in sensitivity map estimation where combining data from neighboring six cardiac cycles may be inaccurate during rapid signal changes. Dark rim artifacts around LV are seen on most of the volunteer images, presumably due to low spatial resolution [22]. The extent of these rims may be reduced by improving the spatial resolution, which requires acceleration of higher rate. Potential techniques for higher acceleration are described in the Discussion section.

**Figure 6:**
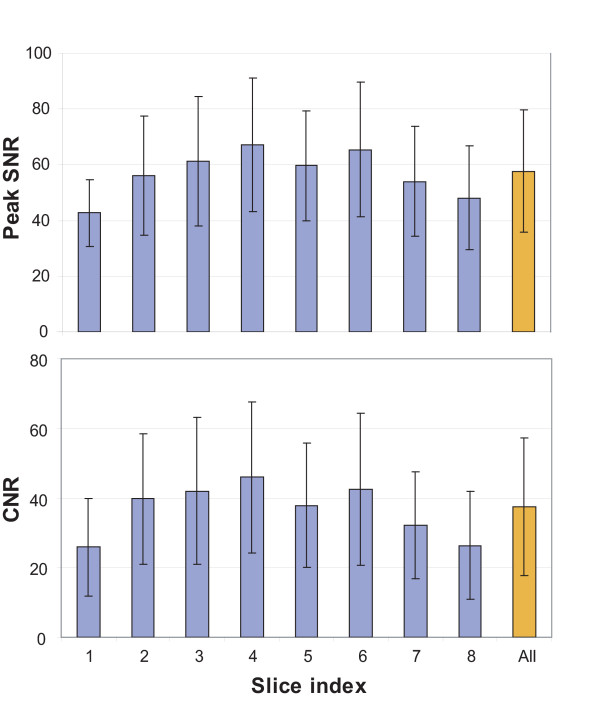
**SNR at peak myocardial enhancement and CNR values for the first eight slices**. The SNR and CNR were 57.6 ± 22.0 and 37.5 ± 19.7 across all segments. Note that the two values are higher in slices at center than slices at edges due to the slab excitation profile of parabolic shape.

Figure 6 shows SNR at peak myocardial enhancement and CNR values in the first eight slices. A total number of 12~18 segments (number of volunteers (3) × number of segments per slice (4 to 6)) were used to compute the average and standard deviation of SNR and CNR values in each slice. Collectively, the SNR and CNR were 57.6 ± 22.0 and 37.5 ± 19.7 across all segments. Note that the two values are higher in slices at center than slices at edges due to the slab excitation profile of parabolic shape.

**Figure 7:**
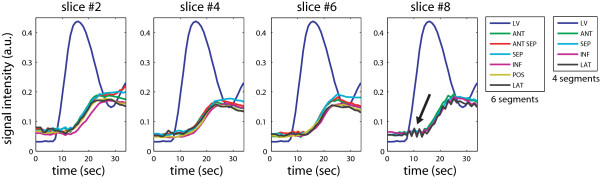
**Time intensity curves in a healthy volunteer**. Representative time intensity curves (TIC) are shown from the 2^*nd*^,4^*th*^,6^*th *^and 8^*th *^slices on a segment basis. Six segments were used for the 2^*nd*^,4^*th *^and 6^*th *^slices (basal to mid-short axis levels), and four segments were used for the 8^*th *^slice (apical level). All TICs show homogenous myocardial enhancement, consistent with normal perfusion. Flickering artifacts caused by errors in coil sensitivity map estimation were observed (see an arrow in the 8^*th *^slice). The artifacts are most severe at the start of LV enhancement, but have no significant effect on the TIC upslope.

**Figure 8:**
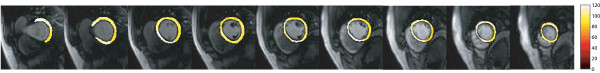
**Colormap visualization of time-intensity curve upslope in a healthy volunteer**. Upslope value of time intensity curve was computed by linear fitting of the curve during signal enhancement, and visualized by a color map. The average upslope value of the whole myocardium is scaled to 100%.

Figure 7 shows representative segment-based TICs obtained from the 2^*nd*^, 4^*th*^, 6^*th *^and 8^*th *^of ten total slices in a volunteer. All TICs show homogeneous myocardial enhancement, consistent with normal perfusion. Flickering artifacts caused by the errors in sensitivity map estimation, manifest as fluctuations in the TIC, as denoted by an arrow. The artifacts are most severe at the start of LV enhancement, but have no significant effect on the TIC upslope. Figure 8 depicts the regional TIC upslope from the first nine slices. The average upslope value of the whole myocardium is scaled to 100% in the map. Only small variation of upslope values was seen over the whole myocardium.

## Discussion

The heart phantom study has demonstrated the superiority of 3D CMR MPI over 2D multi-slice CMR MPI in sizing perfusion defects. The limitation of this phantom study was that only transmural defects were tested, due to the lack of a rubber model for a subendocardial defect. The capability of sizing various types of defects will be investigated by perfusion scans in patients with known coronary artery disease (CAD).

When viewed in a video format (Additional files 1, 2 and 3), the in-vivo perfusion images showed flickering artifacts due to errors in sensitivity map used for SENSE reconstruction. This artifact appeared only during the start of RV and LV enhancement, which barely affected the upslope of myocardial TIC. The flickering can be reduced by employing variable density *k*-space undersampling. The artifacts will be reduced and diffused over the FOV by taking central region of *k*-space only from current cardiac cycle, and combining outer part of *k*-space from neighboring cardiac cycles. More actively, strong quadratic regularization can be used for the separate reconstruction of each coil map from a smaller number of neighboring *k*-space data sets, which will involve less signal change in blood pools.

Six-fold acceleration was achieved by applying SENSE in the in-vivo studies, but further acceleration will be explored for the following reasons. First, spatial resolution of 3.0 × 4.3~4.5 × 10 mm^3 ^used in this study may be insufficient for visualizing subendocardial defects. Higher spatial resolution may also help to avoid the dark rim artifacts which were seen on most volunteer images. Gibbs ringing is one of critical reasons for the artifacts, and the use of high spatial resolution has been shown to restrict its transmural extent [22,23]. Second, the data acquisition time (304 ms) need to be shortened to avoid potential motion artifacts in subjects with high heart rates. An eight-channel receiver coil was used in this study, and using larger array of coil elements would be one way to increase acceleration rate. Recently introduced compressed sensing concept is another promising source for further acceleration [24]. The addition of a regularization of *l*_1_-norm such as total variation to SENSE reconstruction will enable image reconstruction from more highly undersampled *k*-space data.

SNR in a 3D encoded image varies along the partition encoding direction in proportion to the shape of excitation profile (refer to Fig. 6). With TBW of slab excitation = 4 used in this study, the lowest peak SNR in the basal slice (slice #1) was 64% of the highest SNR in the mid short axis slice (slice #4). Evenly high SNR over partition slices should be desired for consistent diagnostic capacity. Variable-rate selective excitation (VERSE) is a technique that modifies the original RF and gradient waveform such that either peak RF value or total RF duration can be reduced with the slice profile unchanged [25,26]. We will explore the use of VERSE technique to increase TBW of slab excitation RF for a sharper profile without increase in RF duration.

In addition to high SNR and contiguous coverage, the large convenience of image registration is a potential merit of 3D encoding. The registration of time-resolved perfusion images is important for subsequent semi-quantitative and/or absolute quantitative perfusion analysis such as TIC upslope. Typically, the registration of 2D images should be performed by non-rigid methods that allow image deformation to accurately model through-plane motion. Since through-plane motion can be registered as accurately as in-plane motion in 3D images, respiratory motion can be corrected for by relatively simple 3D rigid-body model.

## Conclusion

We have demonstrated that 3D CMR MPI is superior to 2D multi-slice CMR MPI in sizing transmural perfusion defects. Mean/standard deviation of errors in estimating the volume fraction across the seven defects were -0.44/1.49%, 2.23/2.97%, and 2.59/3.18% in 3D, 2D 4-slice, and 2D 3-slice imaging, respectively. We have also demonstrated the feasibility of in-vivo 3D MPI at 3T accelerated by sensitivity encoding. 3D CMR MPI at 3T can provide complete LV coverage with excellent image quality and high peak SNR and CNR. Evaluation of this technique in patients with known CAD, and the use of regularized parallel imaging reconstruction for higher spatial resolution and speed, are currently under investigation.

## Competing interests

The authors declare that they have no competing interests.

## Authors' contributions

TS designed and implemented the pulse sequences, performed the phantom and in vivo experiments, performed all data analysis, and drafted the manuscript. HHH contributed to the image reconstruction methods and performance of the phantom study. GMP provided initial motivation for this study and clinically-oriented feedback during the development of methods. KSN conceived of the study, participated in its design, and supervised its performance. All authors read and approved the final manuscript.

## Supplementary Material

Additional file 1 3D perfusion movie from the first subject. This video file contains *in vivo *3D perfusion images from the first subject. Click here for file

Additional file 2 3D perfusion movie from the second subject. This video file contains *in vivo *3D perfusion images from the second subject. Click here for file

Additional file 3 3D perfusion movie from the third subject. This video file contains *in vivo *3D perfusion images from the third subject. Click here for file
